# Cytokinin, auxin, and abscisic acid affects sucrose metabolism conduce to *de novo* shoot organogenesis in rice (*Oryza sativa* L.) callus

**DOI:** 10.1186/1999-3110-54-5

**Published:** 2013-08-13

**Authors:** Shiang-Ting Lee, Wen-Lii Huang

**Affiliations:** Department of Agronomy, National Chiayi University, Chiayi City 600, Taiwan

**Keywords:** *Oryza sativa*, Sucrose, Shoot organogenesis, Phytohormones, Callus

## Abstract

**Background:**

Shoot regeneration frequency in rice callus is still low and highly diverse among rice cultivars. This study aimed to investigate the association of plant hormone signaling and sucrose uptake and metabolism in rice during callus induction and early shoot organogenesis. The immatured seeds of two rice cultivars, Ai-Nan-Tsao 39 (ANT39) and Tainan 11 (TN11) are used in this study.

**Results:**

Callus formation is earlier, callus fresh weight is higher, but water content is significant lower in ANT39 than in TN11 while their explants are inoculated on callus induction medium (CIM). Besides, the regeneration frequency is prominently higher in ANT39 (~80%) compared to TN11 callus (0%). Levels of glucose, sucrose, and starch are all significant higher in ANT39 than in TN11 either at callus induction or early shoot organogenesis stage. Moreover, high expression levels of *Cell wall-bound invertase 1*, *Sucrose transporter 1* (*OsSUT1*) and *OsSUT2* are detected in ANT39 at the fourth-day in CIM but it cannot be detected in TN11 until the tenth-day. It suggested that ANT39 has higher callus growth rate and shoot regeneration ability may cause from higher activity of sucrose uptake and metabolism. As well, the expression levels of *ORYZA SATIVA RESPONSE REGULATOR 1 (ORR1)*, *PIN-formed 1* and *Late embryogenesis-abundant 1*, representing endogenous cytokinin, auxin and ABA signals, respectively, were also up-regulated in highly regenerable callus, ANT39, but only *ORR1* was greatly enhanced in TN11 at the tenth-day in CIM.

**Conclusion:**

Thus, phytohormone signals may affect sucrose metabolism to trigger callus initiation and further *de novo* shoot regeneration in rice culture.

**Electronic supplementary material:**

The online version of this article (doi:10.1186/1999-3110-54-5) contains supplementary material, which is available to authorized users.

## Background

Totipotency ability in individual plant cells can be regenerated to a whole plant by modulating culture conditions (Reinert, 1959). Many factors influence plantlet regeneration ability; examples are genotypes (Huang et al., 2002; Glowacha et al., 2010), phytohormones (Barreto et al., 2010; Feng et al., 2010; Sun and Hong, 2010; Huang et al., 2012), osmotic requirement (Huang and Liu, 2002; Pan et al., 2010; Park et al., 2011; Huang et al., 2012) and carbon sources (Huang and Liu, 2002; Iraqi et al., 2005; Huang et al., 2006; Feng et al., 2010; Silva, 2010). However, the mechanisms of totipotency are still not clarified.

The callus differentiation pathway involves somatic embryogenesis and organogenesis (Jiménez, 2005). Previous studies indicated that both pathways generate entire plantlets from callus in rice but mainly through organogenesis (Jiang et al., 2006; Huang et al., 2012). However, shoot organogenesis from rice calli derived from immature embryo differ among varieties (Huang et al., 2002; Khaleda and Al-Forkan 2006; Dabul et al., 2009). Indica rice varieties generally are less amenable to shoot organogenesis (Zaidi et al., 2006). However, some indica rice varieties have been used to establish high-regeneration-frequency callus culture (Hoque and Mansfied 2004; Wani et al., 2011). In our previous study, the indica rice Ai-Nan-Tsao 39 (ANT39) was the only one screened from 15 cultivars to have high shoot organogenesis frequency (more than 70%) without the need for extra-osmotic stress treatment during callus induction (Huang et al., 2006).

Exogenous carbohydrates are used as the main energy source for explants because of their heterotrophism. Numerous studies have focused on the effect of the kinds and concentrations of carbohydrate supplemented into media (Iraqi et al., 2005; Feng et al., 2010; Silva, 2010); however, the metabolic pathway during callus induction and shoot organogenesis has rarely been discussed. Sucrose is generally used in plant tissue culture; explants uptake sucrose from the medium and hydrolyze it into glucose, fructose for subsequent metabolism (Amino and Tazawa, 1988; Schmitz and Lorz, 1990; Huang and Liu, 2002). Cell wall-bound invertase (CIN) and sucrose transporter (SUT) are the main routes for sucrose absorption and transportation in higher plants. In rice, CIN is involved in many physiological functions such as grain filling, early seed development and inflorescence differentiation (Hirose et al., 2002; Cho et al., 2005Ji et al., 2005; Wang et al., 2008; Wang et al., 2010). Like CIN, SUT was found related to seed development, stress response, and plant growth (Scofield et al., 2007; Chen et al., 2010; Siao et al., 2011; Siahpoosh et al., 2012). However, the effect of these sucrose metabolism-related genes on callus formation and shoot regeneration in rice are still unknown. Changes in sucrose- or starch-metabolism–related enzyme activities in callus culture were found in *Gossypium hirsutum* (Kavi Kishor et al., 1992), *Daucus caroata* (Tang et al., 1999), *Medicago arborea* (Cuadrado et al., 2001), and *Picea mariana* (Iraqi et al., 2005). In our preliminary studies, cellular starch content at callus induction and glucose content at the early regeneration stage were important factors for shoot regeneration in rice (Huang and Liu, 2002; Huang et al., 2006). However, the signals to trigger carbohydrate metabolism and gene expression of related enzymes in rice callus are still poorly understood.

Plant growth regulators (PGRs) have an important role in cell growth and differentiation. Since the classical finding of auxin and cytokinin (Skoog et al., 1965), many papers have shown the effect of PGRs in tissue culture. Both exogenous and endogenous levels of PGRs are highly related to shoot organogenesis (Yin et al., 2008; Zhang et al., 2008; Feng et al., 2010; Huang et al., 2012). Auxin, cytokinin, and ABA are considered key factors for shoot differentiation in callus culture (Brown et al., 1989; Pernisová et al., 2009; Su et al., 2009; Cheng et al., 2010; Vanneste and Friml, 2009; Zhao et al., 2010). Our previous studies showed high levels of endogenous auxin, abscisic acid, and zeatin in highly regenerable rice callus (Liu and Lee, 1996; Huang et al., 2012). B-type response regulator (B-RR) proteins are positive signal regulators for cytokinin signaling (Müller and Sheen, 2007) and the gene expression could be recognized at the cytokinin level (Mason et al., 2005). The B-RR *ORYZA SATIVA RESPONSE REGULATOR 1 (ORR1)* affects cytokinin signaling in rice (Ito and Kurata, 2006). Similarly, the auxin efflux carrier gene family, *PIN-formed* (*PINs*), are a key factor for auxin polar transport (Petrásek et al., 2006; Wang et al., 2009). *OsPIN1* can be detected in calli (Xu et al., 2006) and is related to root emergence and tillering (Huang et al., 2010; Wang et al., 2009). *PIN* gene expression may represent auxin accumulation (Xu et al., 2006; Huang et al., 2010). Besides, *OsLEA1* is regulated by ABA and represented as the signal of endogenous ABA (Shih et al., 2010).

Many studies have shown the cross-talk between phytohormones and sugar sensing in higher plant. Glucose might be a bridge between carbohydrate and phytohormone signaling (Halford and Paul 2003; León and Sheen, 2003; Roitsch et al., 2003; Hartig and Beck, 2006). However, no reports have discussed the relationship between PGR signaling, carbohydrate metabolism, and shoot organogenesis.

In this study, we used two rice cultivars with variable regenerative ability to compare carbohydrate content and gene expression of sucrose-metabolism–related enzymes during callus induction and shoot organogenesis. We further identified the gene expression patterns of *OsPIN1, ORR1* and *OsLEA1* in at the same cultivation period to clarify the relationship between plant hormone signaling and sucrose metabolism in rice callus. Sucrose metabolism may be an important part of shoot organogenesis and may be triggered by phytohormones signaling.

## Methods

### Plant material, callus induction and shoot regeneration

Primary callus was derived from 12- to 14 day-old immature seeds of two rice cultivars (*Oryza sativa* L.) “Tainan 11” (TN11) and “Ai-Nan-Tsao 39” (ANT39) incubated on callus induction medium (CIM) composed of MS basal medium (Murashige and Skoog, 1962) containing 3% sucrose and 10 μM 2, 4-D (Huang et al., 2012). After 2 weeks, calli were transferred to regeneration medium (RM) composed of MS basal medium plus 10 μM NAA and 20 μM kinetin. Both CIM and RM were cultured at approximately 27°C and 200 μmole photons m^-2^ s^-1^ with a 12-h light/dark photoperiod. Calli were harvested at the 10^th^ and 14^th^ days in CIM and fresh weight was measured. Shoot regeneration frequency (%) was evaluated at week 4 after transfer to RM as (calli number with regenerated plantlets / total calli number) x 100%. The calli with regenerated plantlets higher than 1 cm were calculated. The results were from at least 3 independent experiments.

### Measurements of callus growth and water content

Callus was collected on day 10 and day 14 on CIM. These collected calli were weighted as their fresh weight. Dry weights were obtained from these fresh calli that were dried in a ventilating oven at 60°C for 48 hours. Water content was determined from (Fresh weight - Dry weight / Fresh weight) × 100%. Each data was averaged from at least 5 independent calli.

### Extraction and concentration determination of carbohydrates

Samples were harvested after inoculation for 4, 7, 10, 14 days in CIM and 1, 3, 5 and 7 days in RM. The samples were weighed up to 100 mg and dried in a ventilating oven at 60°C for 48 h, then extracted twice with 80% ethanol. The supernatant and pellet were used for soluble sugars (sucrose and glucose) and starch measurement, respectively (Huang and Liu, 2002). A glucose assay kit (Sigma) was used for glucose content determination. The assay solution was added to the ethanol-extracted supernatant and incubated at 37°C for 15 min, and 2 mL of 12N H_2_SO_4_ was added to stop the reaction. The absorption value of 540 nm was obtained by use of a spectrophotometer (U-2001, Hitachi). Each sample was replicated at least 3 times. For sucrose content, ethanol-extracted solution was hydrolyzed by use of invertase (Sigma) for 1 h then underwent the above glucose-content assay (Huang et al., 2006). The absorption value included both sucrose and glucose, so the glucose content was subtracted from this determination to obtain sucrose content.

The pellet was re-suspended with deionic water and boiled for 20 min for measurement of starch content. Amyloglucosidase mixture (90 mM sodium-acetate, 0.1% NaN_3,_ and 25 units amyloglucosidase, pH 4.6) was added for incubation at 55°C for 2 h (Huang and Liu, 2002). The supernatant was collected after centrifugation and quantified as described above for glucose content measurement.

### RNA isolation and quantitative real-time polymerase chain reaction

Total RNA was isolated from the callus (approximately 100 mg) by the TRIzol reagent method (Invitrogen) and treated with TURBO DNA-*free*™ DNase (Ambion) to remove residual DNA. First-strand cDNA was synthesized from 1 μg total RNA with use of MMLV Reverse Transcriptase (Promega). Quantitative RT-PCR involved the IQ^2^ Fast qPCR System (Bio-Genesis) on the ECO™ real-time PCR machine (Illumina). The gene-specific primers designed from the 3’UTR of rice genes are shown in Table 1. The quantitative RT-PCR program initially started with 95°C denaturation for 5 min, followed 40 cycles by 95°C for 30 sec and 60°C for 30 sec. To quantify the relative expression of genes, 2^-ΔΔCq^ values of the target gene were normalized to that of *ubiquitin (OsUBI)* gene.

**Table 1:** **Primers used for quantitative RT-PCR analysis**

Gene	Accession number	Forward (5′-3′)	Reverse (5′-3′)
*OsCIN1*	AY578158	TACGGCAACTTCTACGCATC	CTTGTCGTAGGTGACGCTGT
*OsSUT1*	D87819	TCCTCTGGTTCCACAAACAA	ATTTGCACAAGCTTCACAGC
*OsSUT2*	AB091672	ATTCCCGTTCACCGTTACTC	AGGATTGAGGCTCTTGCACT
*OsPIN1*	AF056027	AACCCGAACACCTACTCCAG	CATCTCGAAGTTCCACCTGA
*ORR1*	AB246780	GCCATTTGCAGAAGTTCAGA	AAGTCCTCCCAGTGAGCCTA
*OsLEA1*	AK064055	GACGACAAGATGCTCAAGGA	CATGCACATGGATACACCAA
*OsUBI*	D12629	CGCAAGTACAACCAGGACAA	TGGTTGCTGTGACCACACTT

### Statistical analysis

Data were analyzed by use of Fisher’s least significant difference (LSD) test with SPSS v 12.0 for Windows (SPSS Inc., Chicago, IL). P<0.05 was considered statistically significant.

## Results

### Rice cultivars differ in callus growth, morphologic features and regeneration frequency

**Figure 1:**
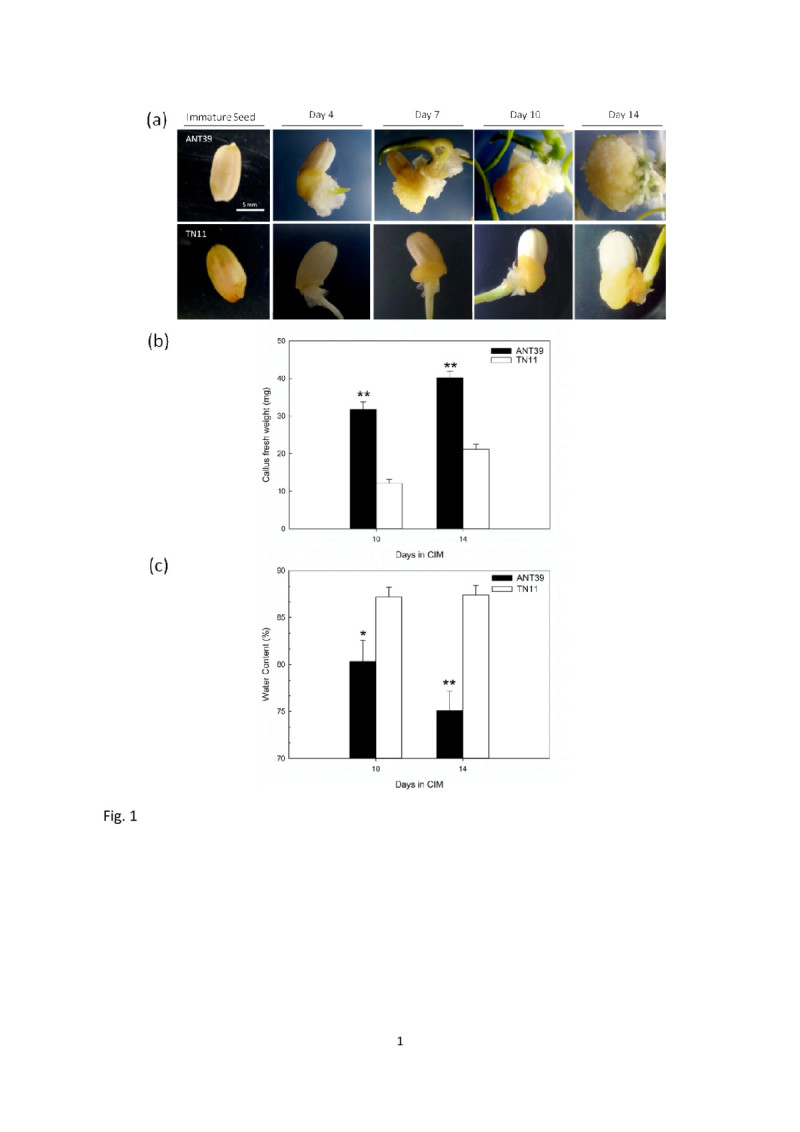
**Morphological features (a), fresh weight (b), and water content (c) of Ai-Nan-Tsao 39 (ANT39) and Tainan 11 (TN11) calli from immature seeds inoculated on MS basal media containing 3% sucrose and 10 μM 2,4-D.** * and ** denote significant difference between ANT39 and TN11 based on the level of 0.05 and 0.01, respectively. Data are mean ± standard error (n=3). Scale bar = 5 mm.

To compare the callus induction and shoot organogenic ability between two rice cultivars, we observed the callus morphology and fresh weight variation. It showed that the calli formed from ANT39 immature seed inoculated on CIM for 3 to 4 days but recognized callus clear from TN11 explant late to days 6 to 7 (Figure 1a). ANT39 calli are large, compact and whitish, but TN11 calli are small and yellowish. The fresh weight of callus was greatly increased and significantly higher in ANT39 than in TN11. The average fresh weight each callus at the fourteenth-day is approximately to 40 mg in ANT39, however, only 22 mg in TN11 at the same day (Figure 1b). On the other hand, the water content at the fourteenth-day callus in ANT39 is 75% but it is approximately to 88% in TN11 callus (Figure 1c). The results suggested ANT39 has higher callus formation ability and growth rate compared to TN11.

**Figure 2:**
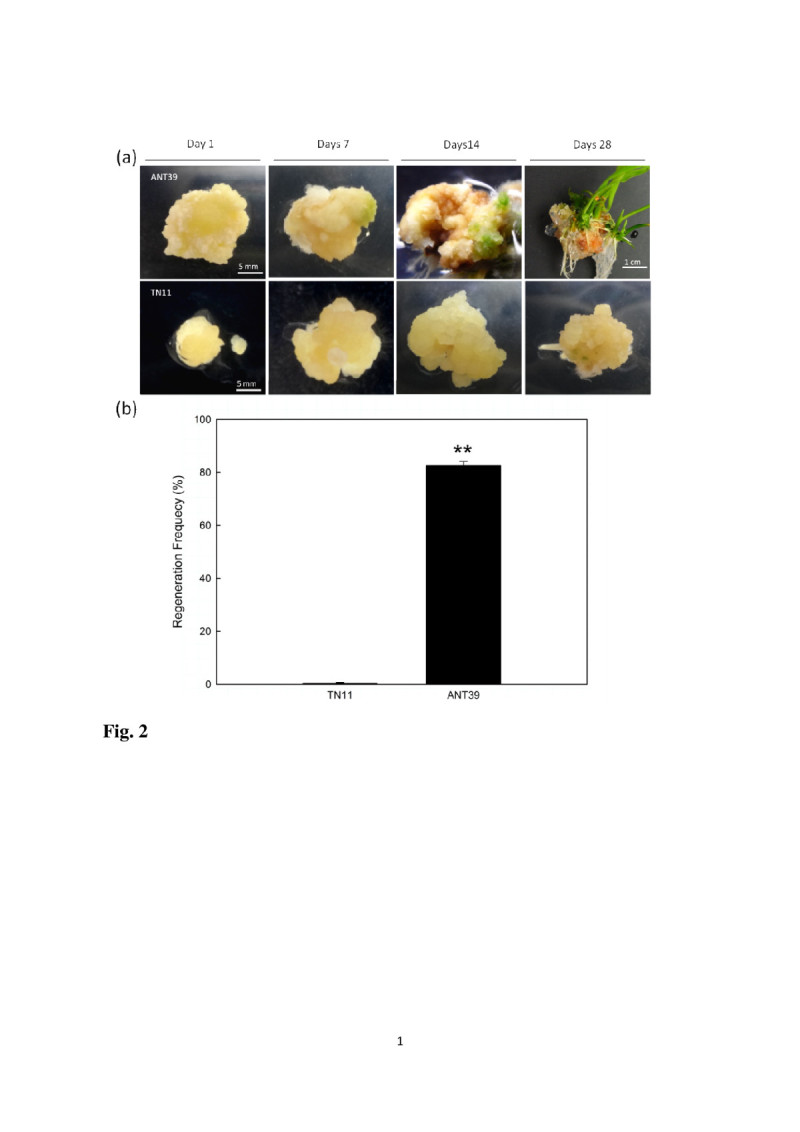
**Shoot organogenesis of 14-day-old callus transferred to regeneration media for 4 weeks. (a)** Morphology of ANT39 and TN11 callus during shoot organogenesis. **(b)** Shoot regeneration frequency. Plantlets higher than 1 cm were recorded. Data are mean ± standard error (n=3). ** denotes significant difference between ANT39 and TN11 based on the level of 0.01.

After transfer to RM, the green spots observed at day 7 in ANT39 calli and continuously spread out, especially at the side contact with the media. The multiple shoots could be emerged after 3 weeks in RM and whole plantlets are further growth (Figure 2a). The shoot organogenic frequency of ANT39 is approximately to 80% (Figure 2b). In contrast, TN11 calli are continue to growth and have no shoot regenerated in RM. Several calli of TN11 only emerged green spots and adventitious roots at late regeneration stage (Figure 2a). In this study, ANT39 and TN11 belong to the highly regenerable (HR) and non-regenerable (NR) cultivar, respectively. ANT39 is the only one cultivar we surveyed in rice possess high shoot regeneration ability without extra osmotic stress treatment during callus induction (Huang et al., 2002). It is suitable used to clarify the possible mechanism with respect to shoot regeneration in rice.

### Relation of carbohydrates content and shoot organogenesis ability

**Figure 3:**
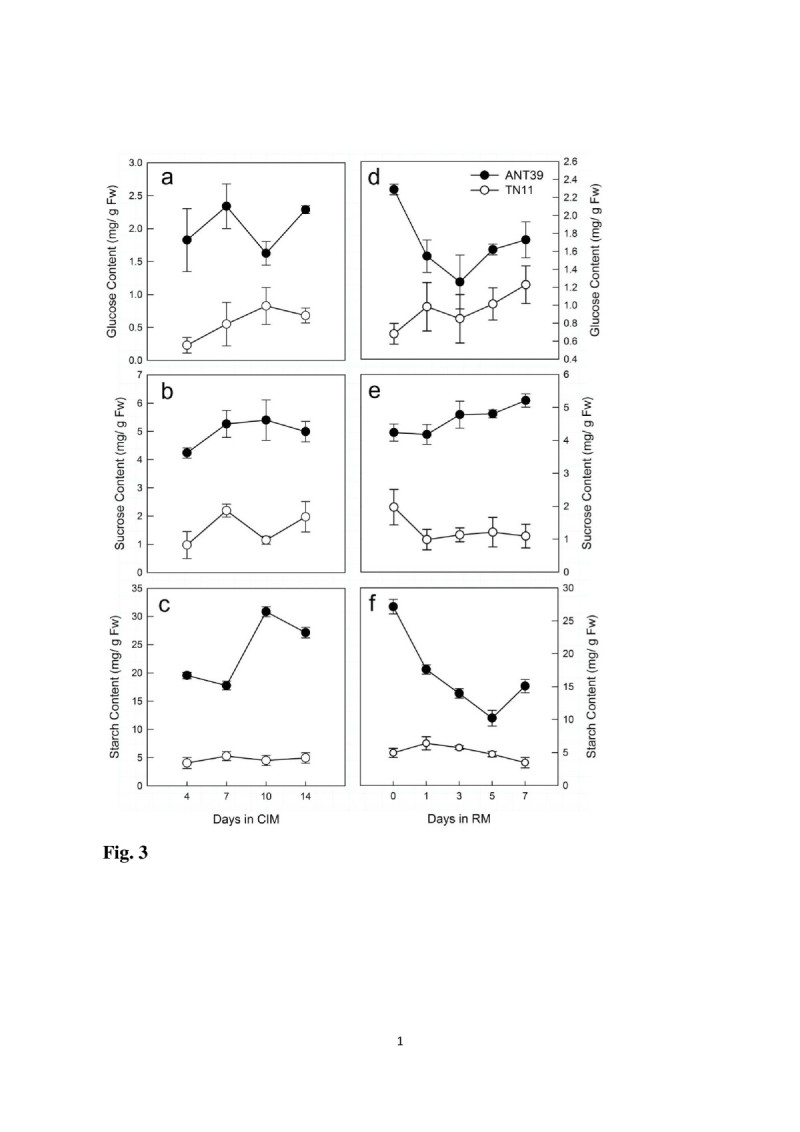
**Carbohydrate content during callus induction and early shoot organogenesis in rice.** Glucose, sucrose and starch content in ANT39 and TN11 calli inoculated in callus induction media **(CIM; a-c)** and regeneration media **(RM; d-f)**. Data are mean ± standard error (n=3).

To clarify the relationship between shoot organogenesis ability and carbohydrate metabolism, glucose, sucrose and starch contents were determined at callus induction and early shoot regeneration stage. The result showed that glucose, sucrose, and starch contents are all significant higher in the HR calli, ANT39, than in NR calli, TN11, either at callus induction or regeneration period (Figure 3). The high carbohydrate content in ANT39 was maintained during callus induction (Figure 3a-c), but the levels of glucose and starch are gradually decreased after transferred to RM in 7 days (Figure 3d, f). All glucose, sucrose, and starch contents in TN11 calli are low and have no significant change during the whole evaluation time. The carbohydrate utilization is higher in ANT39 than in TN11 calli would supply to the energy and osmotic requirement of callus formation and starch accumulation. ANT39 callus possesses high level of starch mainly caused from higher biosynthetic activity and is also observed in our previous study by histochemical analysis (Huang et al., 2006). Besides, high levels of cellular carbohydrates associated with shoot organogenesis in rice callus are similar to the regeneration system induced by osmotic stress (Huang and Liu, 2002).

### Expression of sucrose metabolic enzymes in rice calli

**Figure 4:**
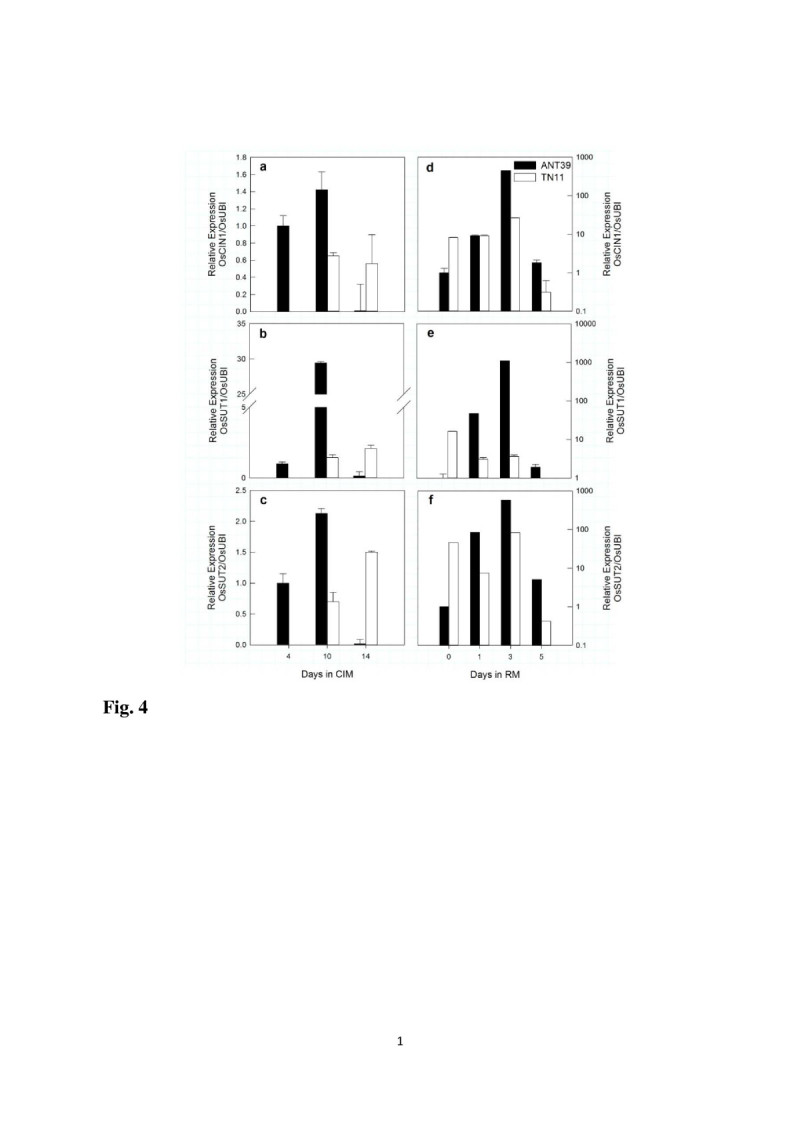
**Real time-PCR analysis of mRNA levels of** ***OsCIN1*** **,** ***OsSUT1*** **and** ***OsSUT2*** **in rice immature seeds during callus induction and early shoot organogenesis. (a-c)** *OsCIN1*, *OsSUT1* and *OsSUT2* levels at days 4, 10 and 14 in CIM. The levels were normalized to that at day 4 in ANT39. **(d-f)** *OsCIN1*, *OsSUT1* and *OsSUT2* levels at days 0, 1, 3 and 5 in RM. Levels were normalized to that at day 0 of ANT39 in RM. *Ubiquitin* level was used as a reference. Data are mean ± standard error (n=3).

According to changes in carbohydrate contents, the efficiency of sucrose uptake and hydrolysis from media is related to calli growth and cell differentiation. We thus determined the mRNA expression of *OsCIN1* and *OsSUTs* during callus induction and early shoot regeneration. *OsCIN1* expression was high in ANT39 from days 4 to days 10 with CIM inoculation then greatly decreased (Figure 4a). The higher expression patterns of *OsSUT1* and *OsSUT2* were similar to *OsCIN1* in ANT39 during callus induction (Figure 4b, 4c). Conversely, these genes did not detected in TN11 calli until days 10 inoculated on CIM; their expression remained high continue to days 14 (Figure 4a-c). These results are identical to the determination of sucrose and glucose contents.

In ANT39 calli, after being transferred to RM, *OsCIN1* expression was significantly induced at the first day and greatly increased at the day 3 (almost 1000-folds). After that, the expression gradually decreased. Like the expression of *OsCIN1*, that of *OsSUT1* and *OsSUT2* was up-regulated at the days 1 and days 3 in RM, then decreased quickly at the days 5 (Figure 4d-f). In contrast, in NR calli, TN11, the expressions of *OsCIN1* and *OsSUT2* were steady in RM, and reduced expression of *OsSUT1* at the days 1. Thus, regardless of callus induction or shoot regeneration stage, ANT39 showed high efficient sucrose uptake and utilization, which would explain the faster callus formation and shoot organogenesis in ANT39 than in TN11.

### Cytokinin, auxin, and ABA signals are related to sucrose uptake

**Figure 5:**
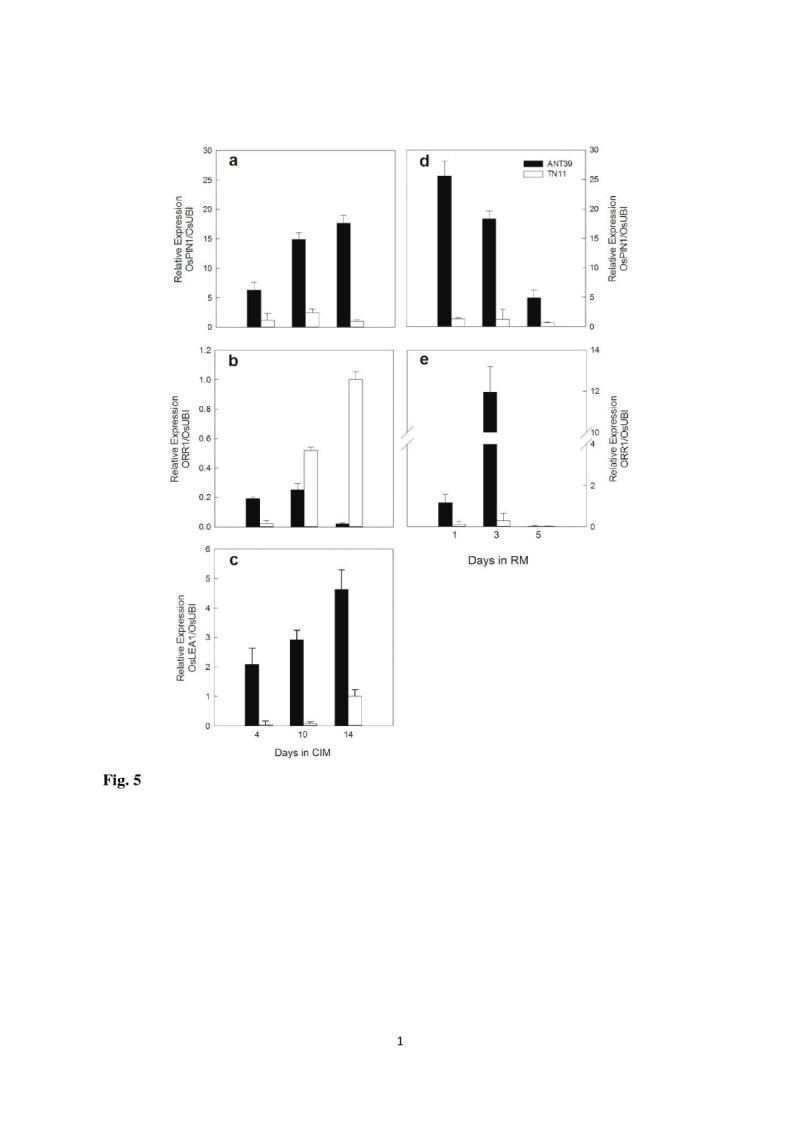
**Real time-PCR quantification of mRNA levels of** ***OsPIN1*** **,** ***ORR1*** **and** ***OsLEA1*** **in rice immature seeds during callus induction (CIM) and early shoot organogenesis (RM). (a, d)** *OsPIN1* levels. **(b, e)** *ORR1* levels. **(c)** *OsLEA1* level. Levels were normalized to that at day 14 of TN11 in CIM. *Ubiquitin* level was used as a reference. Data are mean ± standard error (n=3).

High levels of endogenous auxin, ABA, and zeatin in high regenerable calli are observed in our previous study (Liu and Lee, 1996; Huang et al., 2012). To clarify the relationship between plant hormones and carbohydrate metabolism in rice calli, we determined the expression patterns of the B-type response regulator of cytokinin signaling *ORR1* and auxin efflux carrier *OsPIN1* (Xu et al., 2006), and late embryogenesis-abundant gene *OsLEA1*. *OsPIN1* was highly expressed in ANT39 both at callus induction and shoot organogenesis stages but was low in TN11 and did not change during the whole evaluation period (Figure 5a, 5d). The high expression level of *OsLEA1* is also detected at CIM in ANT39 (Figure 5c). CIM was not supplemented with exogenous cytokinin; thus, the expression level of *ORR1* was the mean endogenous cytokinin level. *ORR1* showed high expression at the early stage in ANT39 calli but higher expression in TN11 at the tenth-day (Figure 5b). After transferred to RM, the expression of *ORR1* was strongly induced in ANT39 on days 1 and 3 because of exogenous kinetin included in the RM. However, the expression of *ORR1* in TN11 did not significant difference during the same regeneration period (Figure 5e). Besides, the expression of *OsLEA1* both in ANT39 and TN11 at early shoot regeneration stage are very low and have no different significantly (data not shown).

## Discussion

Although shoot regeneration and transformation systems are well developed in rice callus, the regeneration frequency is commonly low and is cultivars-dependent (Huang et al., 2002; Khaleda and Al-Forkan 2006; Dabul et al., 2009). Shoot regeneration ability can be greatly enhanced by high osmotic stress treatment during callus induction (Huang and Liu, 2002; Huang et al., 2002; Jiang et al., 2006; Huang et al., 2012). As well, stress-treated calli have lower water content, water potential, and fresh weight but higher glucose and starch contents (Huang and Liu, 2002). ANT39 is a unique cultivar which has high shoot organogenic ability without extra-osmotic stress treatment (Huang et al., 2002). At present study, calli fresh weight was higher but water content lower in ANT39 calli than in non-regenerable TN11 calli (Figure 1b, 1c). No matter of ANT39 or osmotic-induced regeneration system in rice, to initiate the embryogenic calli or organogenic meristemoids at the callus induction stage is the most critical point for further plantlets regeneration (Sugiyama, 1999; Huang and Liu, 2002; Huang et al., 2012).

Carbon sources, phytohormones, and genotypes are well known to affect shoot regeneration in cultured cells. However, the cross-talk among these factors is still little understood. Sucrose is the main carbohydrate supplemented into the culture media and prominently acts as an energy source and osmotic requirement during organogenesis (Verma and Dougall, 1977; Thorpe et al., 1986; Iraqi et al., 2005; Huang et al., 2006; Feng et al., 2010; Silva, 2010). The correlation between starch metabolism and shoot regeneration was also reported in tobacco (Thorpe et al., 1986), sugarcane (Ho and Vasil, 1983), *Begonia* (Mangat et al., 1990) and rice (Huang et al., 2006). However, little is known about the signals to trigger sucrose metabolism in cultured cells, especially at the gene expression level. Here, we found that the possible route from plant hormone auxin, cytokinin and ABA to shoot organogenesis may be through sucrose metabolism in rice callus. Exogenous plant hormones or plant growth regulators used to induce shoot organogenesis interact with endogenous tissue-specific hormones; thus, the level of endogenous hormones in cultured explants and derived calli may be the most important factor in shoot organogenesis. In our previous studies, highly regenerable calli showed high level of endogenous indole-3-acetic acid (IAA) and ABA during whole callus induction; however, high levels of zeatin/zeatin ribosides only showed at 1 week then gradually decreased in CIM. The levels of IAA and ABA quickly decreased and that of zeatin/zeatin ribosides predominantly increased after being transferred to shoot regeneration media (Liu and Lee, 1996; Huang et al., 2012).

ANT39 calli showed high levels of sucrose, glucose and starch (Figure 3). Moreover, the mRNA expression of both *OsCIN1* and *OsSUT2* was significantly induced at the early callus induction stage in ANT39 but it can be detected only at the late stage in TN11 (Figure 4). The high level of cellular sucrose in ANT39 calli may due to direct uptake from media by the sucrose transporter (Figure 4b, 4c). As well, the high glucose content was hydrolyzed by cell wall-bound invertase from sucrose in the culture media (Figure 4a) and from cellular sucrose hydrolysis by soluble invertase and sucrose synthase (Huang et al., unpublished data). However, only a high level of glucose was identified in osmotic stress-treated rice calli, with no difference in sucrose content (Huang and Liu, 2002). The high starch content in ANT39 during callus induction resulted from higher starch biosynthetic enzyme activity but may due to lower degradation activity in osmotic-treated calli (Huang and Liu, 2002; Huang et al., 2006). After transfer to RM, non-regenerable TN11 calli always maintained lower sucrose, glucose and starch contents than that from ANT39 during the evaluated period (Figure 3). However, the content of these carbohydrates were significant higher in ANT39 than in TN11 calli. The higher glucose and sucrose contents may result from uptake through the sucrose transporter and further hydrolysis in ANT39 (Figure 4) but may be hydrolyzed by CIN before sucrose uptake in the osmotic-induced regeneration system (Huang and Liu, 2002). Thus, the signals and carbohydrate metabolism pathway affecting shoot organogenesis in ANT39 calli may differ with osmotic-stress induced regeneration system in rice.

Both exogenous and endogenous plant hormones trigger callus formation and further cell differentiation in plant tissue culture (Barreto et al., 2010; Feng et al., 2010; Pan et al., 2010; Sun and Hong, 2010; Huang et al., 2012). Exogenous plant hormones or plant growth regulators used to induce shoot organogenesis interact with endogenous tissue-specific hormones; thus, the level of endogenous hormones in cultured explants and derived calli may be the most important factor in shoot organogenesis (Huang et al., 2012). How the endogenous hormones signals, especially those of cytokinin, ABA, and auxin, affect cell growth and differentiations are less understood. We determined the expression patterns of *ORR1* as related endogenous cytokinin level (Mason et al., 2005), *OsPIN1* as related endogenous auxin level (Sauer et al., 2006; Tsai et al., 2012) and *OsLEA1* as related endogenous ABA level (Grelet et al., 2005; Shih et al., 2010), respectively. In ANT39 calli, all *ORR1*, *OsPIN1* and *OsLEA1* were strongly induced at the early stage in CIM, but *ORR1* was greatly reduced after days 10 in CIM (Figure 5). In contrast, in TN11 calli, they showed low expressions, except that *ORR1* expression increased after days 10 in CIM. All the expression of phytohormones responsive genes are consistence with endogenous levels of IAA, ABA, and zeatins determined from our previous study (Liu and Lee 1996; Huang et al., 2012). The high levels of endogenous IAA and cytokinin at early callus induction stage in highly regenerable cultivar may response to initiate callus formation and amplification (Skoog et al., 1965). Besides, high level of ABA at late stage in CIM is related to induce organogenic callus formation (Brown et al., 1989; Huang et al., 2012). In addition, the expression patterns of *ORR1* and *OsPIN1* agree with those of *OsCIN1* and *OsSUTs*. Cytokinins and auxins affecting *CIN* and *SUTs* have been found in whole plant systems (Ehness and Roitsch, 1997; Hartig and Beck, 2006; Walters and McRoberts, 2006). Our data showed that both hormones have a similar effect on carbohydrate metabolism during callus induction and shoot organogenesis. In ANT39 regenerable callus, endogenous cytokinin triggered *OsCIN1* and *OsSUTs* gene expression and gained sucrose absorption ability during early callus induction. The expression of *OsCIN1* and *OsSUTs* are strongly inhibited when the auxin transport inhibitor, 2,3,5-triiodobenzoic acid, was supplemented into the CIM (Huang et al., unpublished data ). These expression patterns of sucrose metabolism related genes are positive correlated to the expression pattern of *OsPIN1* gene in CIM but is negative correlated in RM. The reason is high level of IAA response to initiate organogenic or embryogenic competence cell in CIM, however it would be related to root formation at late stage in RM. Besides, the ratio of auxin/cytokinin is higher in CIM may affect the expression of *OsCIN1* and *OsSUTs*. The sucrose absorbed or hydrolyzed in ANT39 callus provided starch biosynthesis as well as energy requirements for cell growth. Our previous histochemical analysis revealed abundant starch granules around whole calli in ANT39 but not non-regenerable rice calli (Huang et al., 2006).

## Conclusions

Finally, we found that plant hormones signaling and carbohydrate metabolism are closely related to shoot organogenesis in rice callus. The explants of the highly regenerable cultivar ANT39 may more sensitive to CIM (MS basal medium containing 3% sucrose and 10 μM 2, 4-D) to quickly increase the levels of endogenous ABA, cytokinin and auxin. The high levels of phytohormones immediately trigger sucrose uptake from the media by a sucrose transporter or cell wall-bound invertase. The high amounts of glucose and sucrose contents provide for callus induction and growth and increase starch biosynthesis. After transferred to RM (MS medium with 3% sucrose, 20 μM kinetin and 10 μM NAA), exogenous kinetin and NAA led to sucrose absorption and hydrolysis to provide the energy to initiate shoot organogenesis and further development. We reveal the association among phytohormones, sucrose metabolism, and shoot organogenesis in rice calli. However, the signal transduction pathways of plant hormones to sucrose and starch metabolism still need to be further determined.

## Authors’ original submitted files for images

Below are the links to the authors’ original submitted files for images. Authors’ original file for figure 1 Authors’ original file for figure 2 Authors’ original file for figure 3 Authors’ original file for figure 4 Authors’ original file for figure 5

## Abbreviations

2: 4-D: 2, 4-dichlorophenoxyacetic acid
ABA: Abscisic acid
CIM: Callus induction medium
CIN: Cell wall-bound invertase
NAA: 1-naphthalene acetic acid
ORR1: ORYZA SATIVA RESPONSE REGULATOR 1
PGRs: Plant growth regulators
PIN: PIN-formed protein
RM: Regeneration medium
SUT: Sucrose transporter.
