# Supplementary figures and images for: Cytokinin, auxin, and abscisic acid affects sucrose metabolism conduce to de novo shoot organogenesis in rice (Oryza sativa L.) callus
Source: Bot Stud. 2013 Aug 13;54:5. doi: 10.1186/1999-3110-54-5 (PMC5383921; doi:10.1186/1999-3110-54-5)

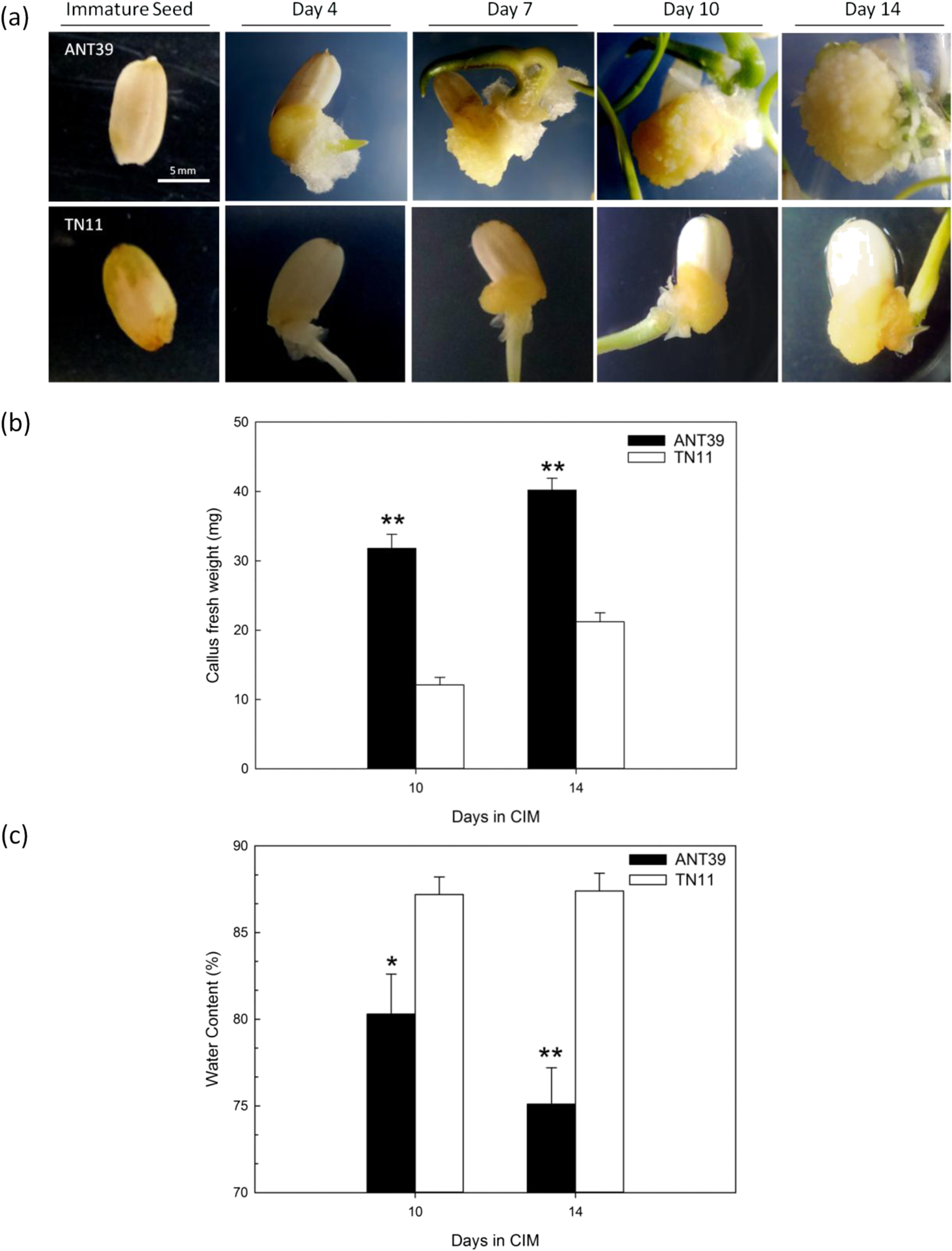

Supplement: Supplementary file 1 — Authors’ original file for figure 1 [file 40529_2013_5_MOESM1_ESM.tif]

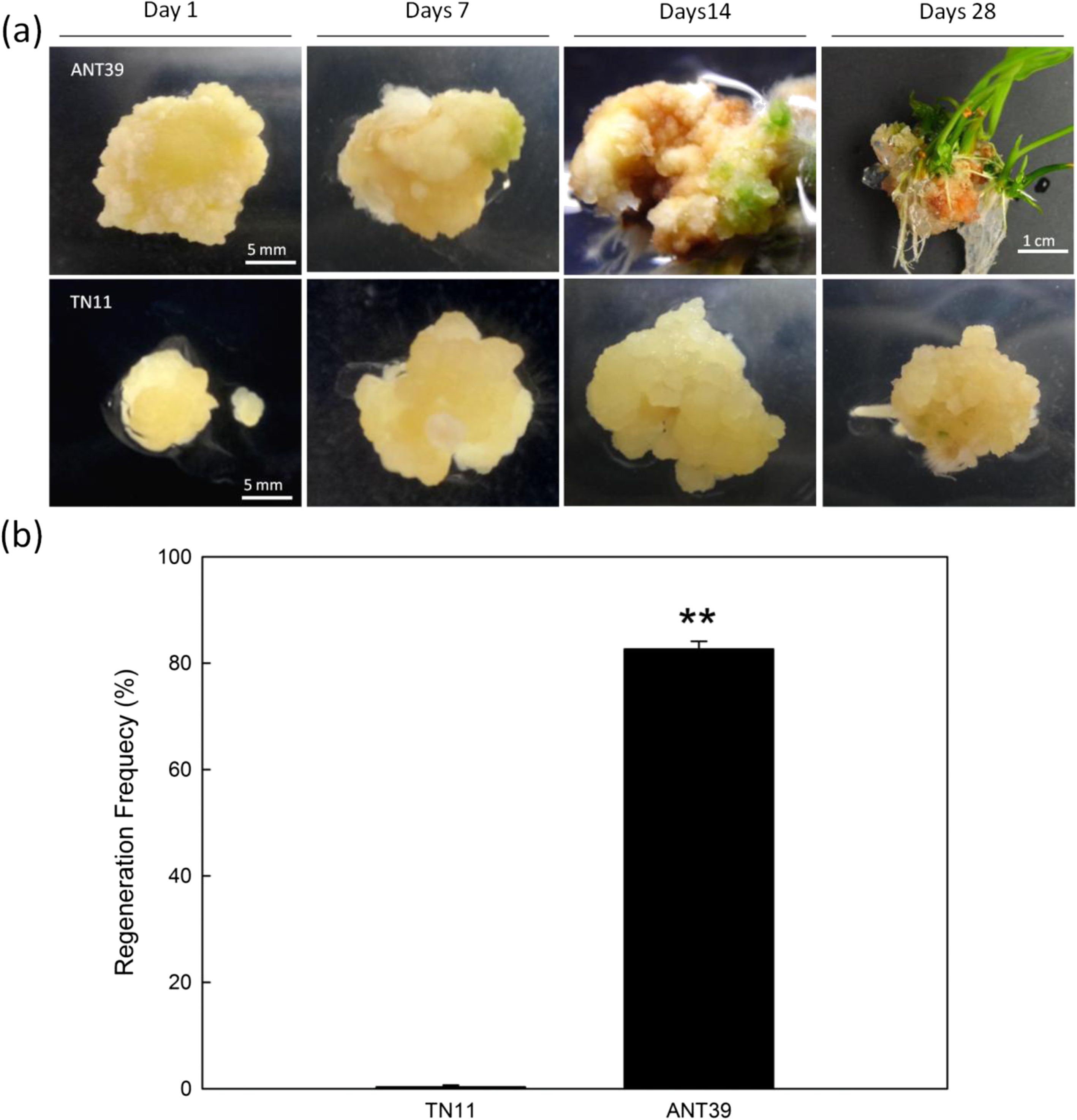

Supplement: Supplementary file 2 — Authors’ original file for figure 2 [file 40529_2013_5_MOESM2_ESM.tif]

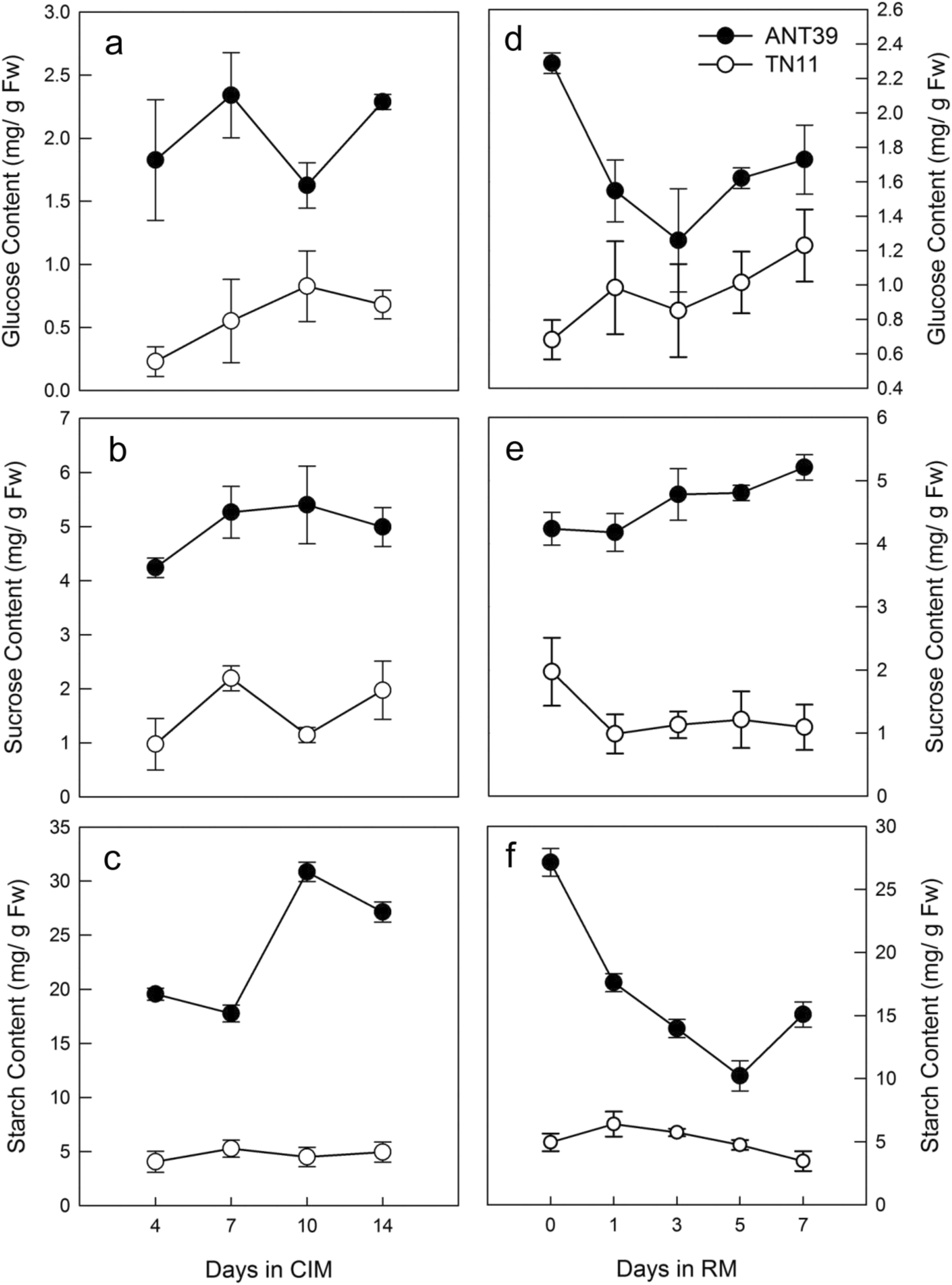

Supplement: Supplementary file 3 — Authors’ original file for figure 3 [file 40529_2013_5_MOESM3_ESM.tif]

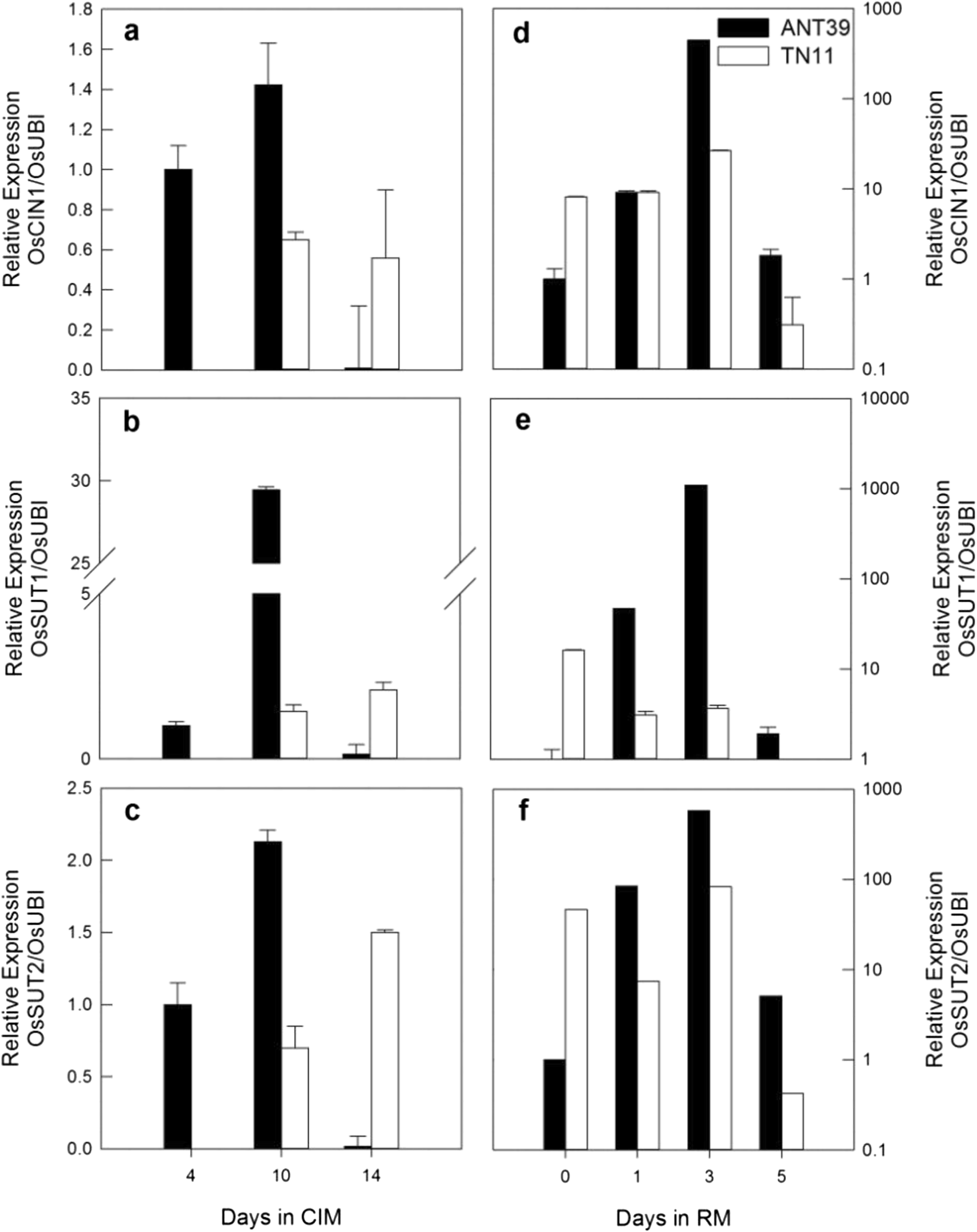

Supplement: Supplementary file 4 — Authors’ original file for figure 4 [file 40529_2013_5_MOESM4_ESM.tif]

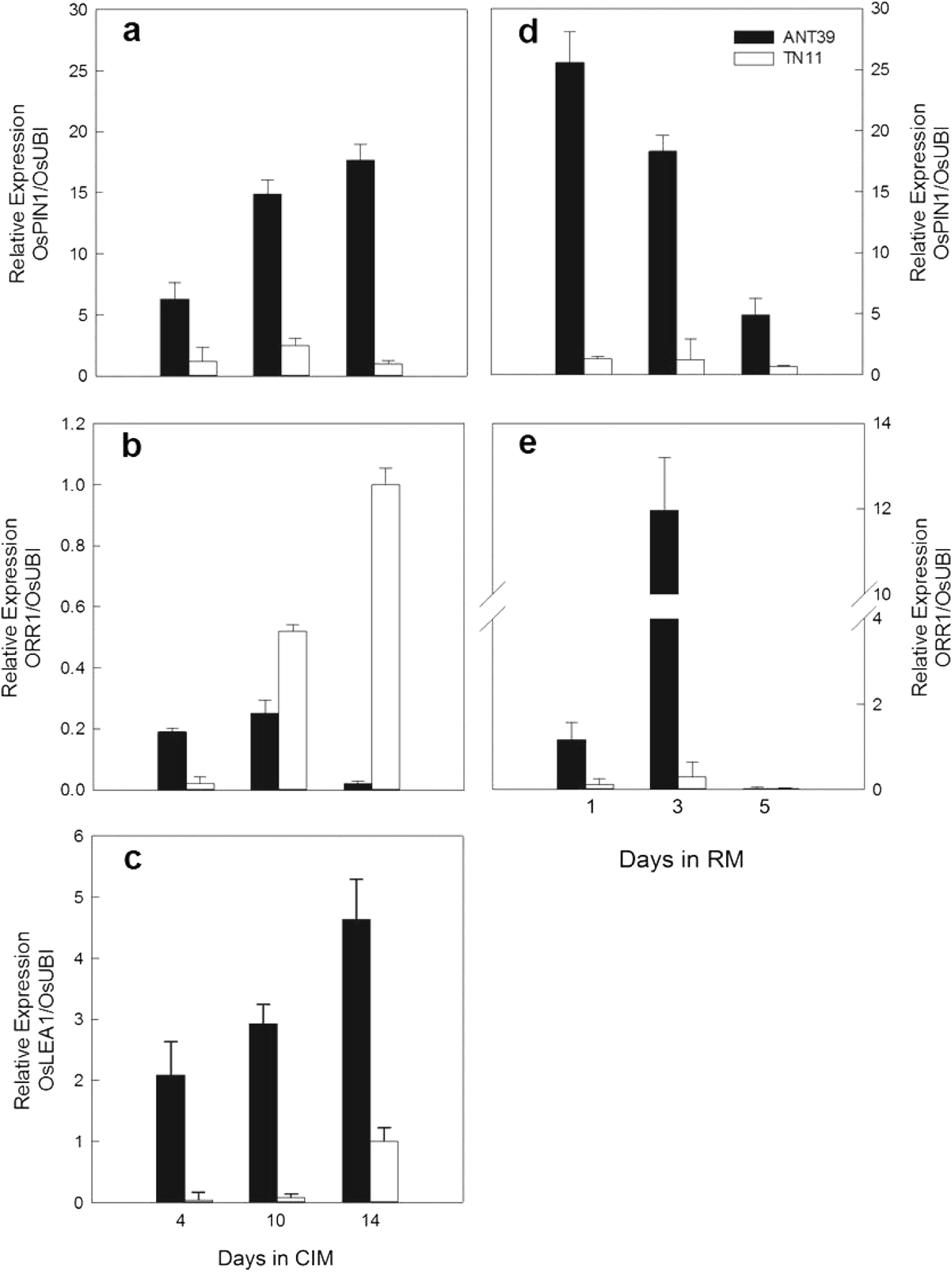

Supplement: Supplementary file 5 — Authors’ original file for figure 5 [file 40529_2013_5_MOESM5_ESM.tif]
